# SWR1 Chromatin Remodeling Complex: A Key Transcriptional Regulator in Plants

**DOI:** 10.3390/cells8121621

**Published:** 2019-12-12

**Authors:** Mohammad Aslam, Beenish Fakher, Bello Hassan Jakada, Shijiang Cao, Yuan Qin

**Affiliations:** 1 Key Laboratory of Genetics, Breeding and Multiple Utilization of Crops, Ministry of Education, Fujian Provincial Key Laboratory of Haixia Applied Plant Systems Biology, College of Agriculture, Fujian Agriculture and Forestry University, Fuzhou 350002, China; beenishfakher@icloud.com (B.F.); bellojakada@gmail.com (B.H.J.); csjiang1123@126.com (S.C.); 2 State Key Laboratory for Conservation and Utilization of Subtropical Agro-Bioresources, Guangxi Key Lab of Sugarcane Biology, College of Agriculture, Guangxi University, Nanning 530004, China; 3 College of Life Sciences, Fujian Agriculture and Forestry University, Fuzhou 350002, China; 4 College of Forestry, Fujian Agriculture and Forestry University, Fuzhou 350002, China

**Keywords:** chromatin remodeling complex, SWR1-C, H2A.Z, flowering time regulation

## Abstract

The nucleosome is the structural and fundamental unit of eukaryotic chromatin. The chromatin remodeling complexes change nucleosome composition, packaging and positioning to regulate DNA accessibility for cellular machinery. SWI2/SNF2-Related 1 Chromatin Remodeling Complex (SWR1-C) belongs to the INO80 chromatin remodeling family and mainly catalyzes the exchange of H2A-H2B with the H2A.Z-H2B dimer. The replacement of H2A.Z into nucleosomes affects nucleosome stability and chromatin structure. Incorporation of H2A.Z into the chromatin and its physiochemical properties play a key role in transcriptional regulation during developmental and environmental responses. In *Arabidopsis*, various studies have uncovered several pivotal roles of SWR1-C. Recently, notable progress has been achieved in understanding the role of SWR1-C in plant developmental and physiological processes such as DNA damage repair, stress tolerance, and flowering time. The present article introduces the SWR1-C and comprehensively reviews recent discoveries made in understanding the function of the SWR1 complex in plants.

## 1. Introduction

In multicellular organisms, cell differentiation and morphogenesis depend on turning on and off of the specific set of genes in different tissues and during different developmental stages. Gene expression requires the coordinated action of RNA polymerase and transcription factors, whose product later determines the fate of the cell in a particular tissue. Inside a cell, gene expression is tightly regulated as the requirement of gene product changes with tissue, developmental stages, and external and internal factors. The regulation of a gene primarily lies in the arrangement of DNA and its interaction with other proteins. Intriguingly, DNA is copiously wrapped in such a way that it can accommodate itself inside the nucleus. Histone proteins act as the spool for the systematic coiling of DNA around them, creating compact chromatin. Structurally, chromatin is a protein–DNA complex with a series of nucleosome units containing two copies of H2A, H2B, H3, and H4 histone proteins with approximately 147 base pairs of DNA enveloped around it [1,2]. This arrangement of chromatin is crucial for the regulation of gene expression during different biological processes, including development and responses to environmental impulses [3]. Suffice to say, for the efficient gene expression, replication, and DNA repair, DNA requires uncoiling to become accessible to these machineries. 

**Figure 1:**
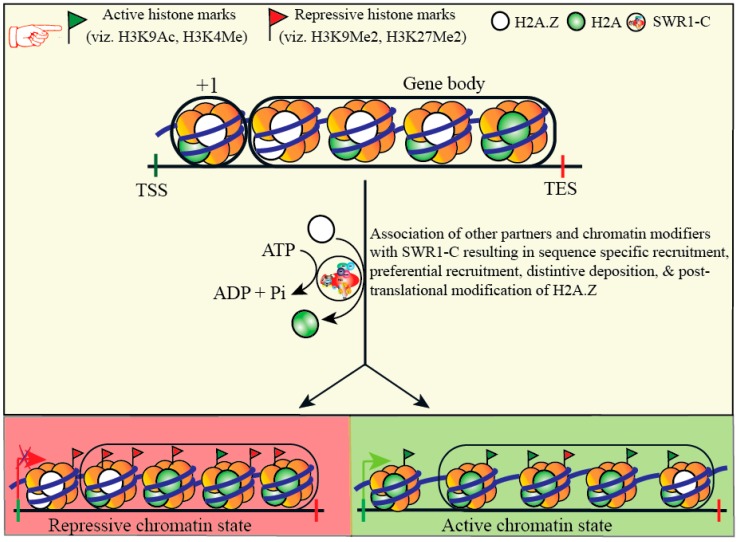
Schematic representation of SWI2/SNF2-Related 1 Chromatin Remodeling Complex (SWR1-C) mediated chromatin remodeling and swapping of H2A-H2B with the H2A.Z-H2B dimer, resulting in repressive and active chromatin state.

Cellular machinery could utilize all or any of the three ways (epigenetic regulation) to untie nucleosomal restraints: a) Alterations in N-terminal tails of histones by phosphoryl-, acetyl-, methyl-, and ubiquityl moiety, which could work as a signal for other nuclear machinery [4]; b) employment of histone variants specific to four core canonical histones that could replace the latter to intervene in their structure and behavior [5]; c) the energy-driven shift of nucleosomal apparatus using ATP [6,7]. Post-translational histone modifications and ATP-dependent chromatin remodeling regulate the chromatin structure. The process alters nucleosome positioning and arrangement, consequently affecting the DNA accessibility via chromatin spatial arrangement [6]. Chromatin remodeling and spatiotemporal association of genes with different regulatory regions fine-tune the gene expression [8,9]. At the cellular level, the chromatin assembly requires a significant rearrangement to perform a variety of tasks. Chromatin remodeling complexes (CRCs) remodel chromatin in such a way that it releases the tightly wrapped DNA and allows access for transcription to occur, and vice versa. CRCs play a crucial role in the regulation of transcription by changing the composition and controlling the structure of chromatin [10,11]. The CRCs utilize the energy from ATP to displace or change the composition of the nucleosome [12]. All the four major subfamilies of CRCs, namely SWI/SNF, INO80, ISWI, and CHD, use ATP as the source of energy to perform their task [13]. Chromatin remodeler SWR1-C is a member of the INO80 CRC subfamily that exchanges H2A variant, H2A.Z, directly to nucleosomes (Figure 1) [6]. The histone variant H2A.Z, a member of H2A family, is the most conserved histone variant which accounts for approximately 15% of total cellular H2A content [14]. H2A.Z has been studied in several different species including yeast, Drosophila, humans, and plants [15,16,17,18]. In *Arabidopsis,* H2A.Z is encoded by three genes: *HTA8, HTA9, HTA11* [18]. Several findings suggest that the histone variant H2A.Z performs various decisive functions in cellular processes via influencing the chromatin structure (for more details see recent review by Kumar, S.V.) [19]. 

In *Arabidopsis*, SWR1-C is composed of several proteins, including ACTIN-RELATED PROTEIN 6 (ARP6; also known as SUPPRESSOR OF FRIGIDA3 (SUF3) and EARLY IN SHORT DAYS 1 (ESD1)), SWR1 COMPLEX SUBUNIT 6 (SWC6; also known as SERRATED LEAVES AND EARLY FLOWERING (SEF)), YEAST ALL1-FUSED GENE FROM CHROMOSOME 9 (YAF9A and YAF9B), and PHOTOPERIOD-INDEPENDENT EARLY FLOWERING1 (PIE1) [20,21,22,23]. Increasing evidence suggests that SWR1-C is associated with the response to cellular stress like osmotic stress, phosphate starvation, and biotic stress (Table 1, Supplementary Figure S1) [19,20,24,25]. Alongside this, the role of SWR1-C in floral transition and thermosensory responses is well established [26,27,28]. Further, it is observed that the mutations in the chromatin-remodeling components compromise the plant’s ability to perform key tasks essential for growth and development [21,22,29]. Herein, we have introduced the SWR1-C chromatin remodeling complex and reviewed recent progress made in understanding the function of SWR1-C during developmental processes and response to environmental cues in plants.

## 2. A Comparative Analysis of SWR1-C

SWR1-C, exclusive for trading H2A-H2B dimer with H2A.Z-H2B dimer, was first reported in yeast and has a profound effect on the development of budding Yeast [6,13]. It is a unique ATPase-containing Swi2/Snf2-related multiprotein CRC with approximately 14 subunits (Table 2) [6,13,41]. The ATPase Swr1 works as a scaffold and contributes to the enzymatic activity of the complex. The non-catalytic subunits of SWR1-C including ARP6 and SWC6 are also crucial for histone swapping. ARP6, SWC6, and SWR1 COMPLEX SUBUNIT 2 (SWC2) proteins are mutually obligatory for association and activity of the complex and act as a sub-complex within SWR1-C [42,43]. The accessory proteins complete the complex by associating with each other, and a significant depletion in H2A.Z is observed in the loss of function mutants of any of these proteins [42]. Mutational analysis of some non-integral SWR1-C components indicate that in vivo deposition of H2A.Z to chromatin is dependent on ARP6, YAF9, SWC2, and SWC6 together with SWR1 ATPase [43,44]. Few of these proteins are also shared between other CRCs such as INO80 complex and the yeast Nucleosome Acetyle-transferase of histone 4 (NuA4) complex. NuA4 complex also regulates the exchange of H2A.Z in cooperation with SWR1-C suggesting their additional role beyond SWR1-C core functions [42,45].

In mammals, the multi-subunit complex SNF2-related CREB-binding protein activator protein (SRCAP) is known as the structural and functional homolog of yeast SWR1-C. SRCAP complex possesses 11 subunits which are similar to yeast SWR1-C. SRCAP exchanges H2A-H2B with H2A.Z-H2B in the nucleosomes of higher eukaryotes [46,47]. Similar to NuA4 complex in yeast, mammals also possess 60 kDa Tat-interactive protein (TIP60), a multi-subunit protein complex, with the ability to mediate the H2A.Z deposition [48,49]. 

Experimental evidence from several genetic and biochemical studies suggest that plants also possess one or more homologs of yeast SWR1 and animal SRCAP complexes subunits [31,50]. The presence of SWR1-C subunits including *ARP6*, *SWC2*, *SWC6*, *PIE1,* and H2A.*Z* encoding genes (*HTA8*, *HTA9*, *HTA11*) indicate that the SWR1-C is conserved in plants [21,22,32,51,52]. Moreover, two subunits of SWR1-C, SWC6, and YAF9a, have been validated for their interaction with SWC4 in a pull-down assay in *Arabidopsis* [40]. Additionally, the direct interaction of PIE1 with other subunits such as ARP6, SWC6, and HTA8/9/11 is also established [31,52]. Mutations in *Arabidopsis* SWR1-C subunit genes result in pleiotropic phenotypic alterations including serrated leaves, altered petal number, early flowering, reduced fertility, and loss of apical dominance [21,22,23,29,31,51]. Consistently, SWR1-C regulates the gene expression during meiosis, as seen in *Arabidopsis arp6* mutants. These mutants display abnormality during prophase I of meiosis [30,53]. However, *pie1* mutants display a more severe phenotype compared to *arp6*, *swc6*, and *hta9;* with *hta11* plants indicating that PIE1 may have additional functions beyond H2A.Z deposition by SWR1-C [31,54,55]. Additionally, PIE1 works as scaffold for other subunits to associate and form the active complex and it may be possible that an assembled but inactive complex in *arp6* and *swc6* mutants has a different impact on gene expression compared to a completely absent SWR1-C complex in a *pie1* mutant. Recently, two groups independently identified the conserved subunits of yeast SWR1 and SRCAP complexes in *Arabidopsis* using ARP6 subunit as bait for IP-mass spec and Tandem Affinity Purification (TAP) [56,57]. They also identified additional interacting partners associated with ARP6, including the plant homeodomain (PHD)- and Bromo domain-containing protein Methyl CpG-BINDING DOMAIN 9 (MBD9). The findings from both studies suggest that the MBD9 interacts with the *Arabidopsis* SWR1-C and plays a crucial role in H2A.Z deposition at active genes [56,57]. Consistently, MBD9 interaction with SWR1-C is also shown in a forward genetic screen for REPRESSOR OF SILENCING 1 (ROS1) mediated DNA demethylation and anti-silencing [9]. Nie et al. found that the SWR1-C subunits ARP6 and PIE1 restrict transgene silencing by reducing the DNA methylation in association with MBD9 and nuclear protein X1 (NPX1). These studies collectively establish that the plants also possess a bonafide homolog of yeast SWR1-C and SRCAP complexes. However, much work is needed to examine the role of each subunit in plant SWR1-C to have a better understanding of their function. 

## 3. SWR1-C in DNA Damage Repair

Being sessile, plants are regularly antagonized by environmental factors and metabolic derivatives that could cause DNA damage. Genotoxic stress and free radicals produced during cellular metabolism, along with environmental insults like ionizing radiation (IR) and toxic chemicals, incite double-strand breaks (DSBs) of DNA [58]. Damage in DNA must go for repair to ensure the transmission of correct genetic information, if not it could be fatal to the cell [30]. Chromatin remodeling is an essential step during DNA repair and various components of CRCs have been associated with DNA damage repair in *Arabidopsis* [30,59,60]. During the process of repair, the Mre11–Rad50–Xrs2 (MRX) complex collaborates with other agents and increase the production of single-stranded DNA, a process known as resection. DNA damage checkpoint signaling process is then coordinated by Mec1 kinase that localizes at the damaged DNA sites along with other checkpoint factors [61]. The SWR1-C accomplishes DNA damage repair generally by two different methods, either by the remodeling of chromatin structure or by the posttranslational alteration in histone tail residues [62]. Defects in DSB repair in mutants of the INO80 complex of plants and mammals indicate a conserved mechanism of the INO80 complex during repair pathway, however, the *S. cerevisiae* SWR1 ATPase subunit participates in the error-free non-homologous end joining (NHEJ) pathway [63,64]. Mutational studies have provided an insight into the role of CRCs in repair of the double-strand break of DNA. Mutations in SWR1-C subunits increase the sensitivity to DNA damage, meiotic abnormality, and decrease somatic homologous recombination (SHR) [30]. Consistently, the mutation of SWR1-C subunits *pie1, arp6,* and *swc6* results in hypersensitivity against various DNA damaging agents [30]. Collectively, it is evident that in addition to transcriptional regulation, SWR1-C is also essential for genome stability as it plays the key role in DNA replication, DNA repair, and the restoration of the chromatin organization post repair process [65,66].

## 4. SWR1-C Involvement in Plant Stress Response

Plants display several adaptive strategies during their encounters with unfavorable conditions, including the expression of stress-responsive genes [67,68]. Moreover, during the response to environmental and developmental signals, a plant cell could generate several epigenomes from its genome [58,69]. Additionally, heterochromatin’s structural rearrangement is also crucial during different types of stress. The nuclear organization change against stress conditions could be imperative for the functional response. Recently, several reports have established the importance of the SWR1-C during the plant stress response [20,24,25,52]. However, the functions of SWR1-C during the response to other stress are still obscure. During the stress condition, SWR1-C controls the expression of stress-responsive genes by positively and/or negatively regulating them. Interestingly, loss of mutants of subunits *pie1*, *swc6*, *hta9*, and *hta11* displays an impaired response to necrotrophic and biotrophic pathogens, however, *arp6* shows increased resistance to these pathogens [20]. Though the SWR1-C subunits show a differential response in immunity, it is obvious that SWR1-C plays an essential role in the defense response pathway of *Arabidopsis* [20,52]. The involvement of SWR1-C during environmental responses suggests a more complex regulatory network than expected. Additional studies are required to completely understand the role of SWR1-C during stress. 

## 5. SWR1-C in Plant Development

SWR1-C also plays a crucial role in various developmental processes of plants [19]. For instance, *arp6* mutants display defects in chromosome pairing and organization during female meiosis I with reduced seed set [53]. Furthermore, the expression of the transcription factor gene *WRKY28*, which is reported to play the key role in megaspore mother cell (MMC) specification, is activated by the cytochrome P450 (CYP78A5) KLU in conjunction with the SWR1-C. Besides, H2A.Z deposition by SWR1-C at *WRKY28* is dependent on KLU, indicating that the recruitment of SWR1 at *WRKY28* is assisted by KLU. This results in increased expression of *WRKY28* which maintains the identity of megasporocyte and prevents multiple somatic cells from differentiating into megaspore mother cells [37]. 

In higher plants, the vegetative stage and reproductive stage show various phenotypic and physiological differences between them [36]. The phase transition stimuli could be detected by single or multiple genetic pathways and transmitted into the principal regulatory component that governs the phase change [35]. For example, the expression of *SQUAMOSA PROMOTER BINDING LIKE* (*SPL*)s genes, that assist in the vegetative phase change in *Arabidopsis,* is increased due to a decrease in the expression of *MIR156A* and *MIR156C*. A recent study uncovered the role of SWR1-C in the promotion of juvenile phase by *MIR156A* and *MIR156C*. Mutations in SWR1-C subunits *arp6*, *swc6* and H2A.Z encoding genes reduced the expression of *MIR156A* and *MIR156C* resulting in the acceleration of the vegetative phase change [36]. The H2A.Z facilitated deposition of H3K4Me3 increases the expression of *MIR156A* and *MIR156C* early in shoot development, however, *arp6* and *swc6* mutants did not show the acceleration in the temporal reduction in miR156, suggesting that SWR1-C does not regulate the timing of phase change, rather it promotes the juvenile phase [36]. Moreover, SWR1-C in coordination with ERECTA signaling controls the expression of *PACLOBUTRAZOL RESISTANCE1* (*PRE1)* family genes and promotes the pedicel length which retains the inflorescence architecture. SWR1-C incorporates the H2A.Z at the *PRE1* gene family and ERECTA controls the expression of the *PRE1* gene family [38].

## 6. SWR1-C and Flowering Time Regulation

**Figure 2:**
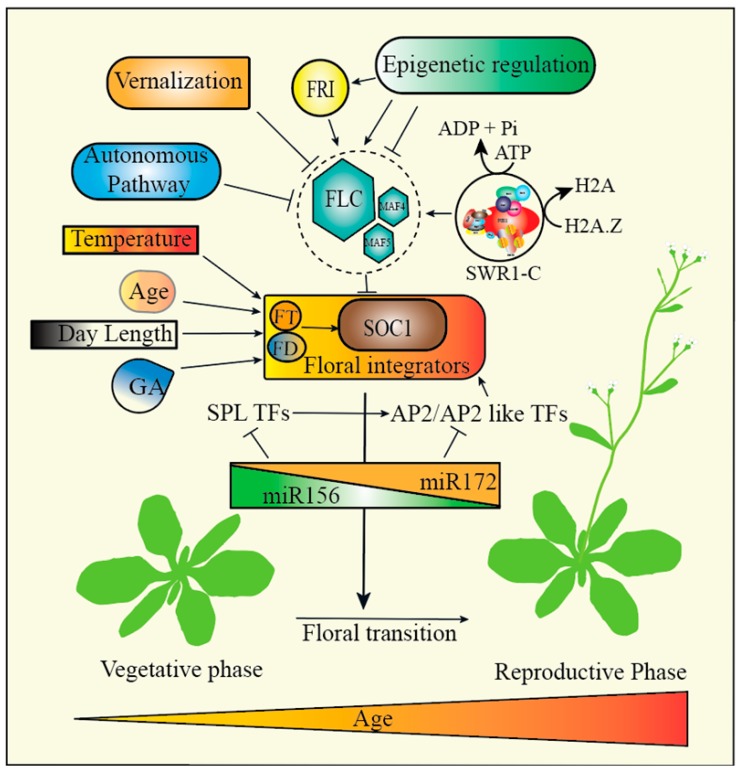
Regulation of flowering time in *Arabidopsis*. *FLOWERING LOCUS C (FLC),* plays a key role in flowering time induction by acting as a repressor of flowering. The expression of *FLC* is regulated by *FRIGIDA*, vernalization, autonomous pathways, and SWR1-C. *FLC* restricts flowering by directly repressing the key genes responsible for flowering including *FLOWERING LOCUS T (FT), FD*, and *SUPPRESSOR OF OVEREXPRESSION OF CONSTANS 1 (SOC1).* The vernalization pathway promotes flowering in response to the prolonged exposure to cold temperature by turning off the *FLC*. Flowering can also be induced by age, photoperiod, and Gibberellic acid (GA). The photoperiod promotes flowering in response to day length. Additionally, miR156/miR172 also play a critical function in phase change by targeting the transcription factors *SQUAMOSA PROMOTER BINDING PROTEIN-LIKE (SPL)* and *APETALA2* (*AP2*) like genes.

The flowering of *Arabidopsis* is under the control of several genetic pathways including photoperiod, vernalization, temperature, and gibberellins (Figure 2) [70]. Moreover, flowering could also happen even in lieu or independently of environmental factors [71]. In *Arabidopsis,* flowering time is regulated by several genes including *FRIGIDA (FRI)*, *FLOWERING LOCUS C (FLC)*, *FLOWERING LOCUS T (FT)*, and *SUPPRESSOR OF OVEREXPRESSION OF CONSTANS 1 (SOC1)* [72,73]. *FRI* encodes a protein that increases the transcript level of *FLC* [74,75]. However, FLC, a repressor of flowering belonging to the MADS-box protein family, negatively regulates the expression of genes required for flowering [76,77,78]. Thus, the mutations in the genes regulating the expression of *FLC* lead to change in the flowering time through the change in the expression of downstream floral pathway integrator genes. Recent studies suggest that the expression of *FLC* is regulated by chromatin remodeling. Several genetic screens led to the identification of *FLC* activators (PAF, SWR1-C complex and FRI-C) [26,74,79,80] and suppressors, such as vernalization insensitive 3 (VIN3) that attenuate the expression of *FLC* [81]. The FRI protein increases the *FLC* expression by forming a large protein complex; FRI-C. FRI-C interacts with chromatin modification factors SWR1-C and EFS to increase the recruitment of SWR1-C and EFS at the *FLC* promoter and regulate *FLC* expression [80]. The recruitment of SWR1-C at *FLC* promoter was also confirmed in a chromatin immunoprecipitation assay where SWC6 and ARP6 bind to the *FLC* promoter [31]. Additionally, in a different assay SWC6 co-localized with ARP6. Consistently, the authors also showed that PIE1, ARP6, SWC6, and SWC2 form a complex, suggesting that the SWR1-C regulates flowering time by modulating *FLC* expression [31]. A significant decrease in the expression of *FLC* gene results in the early flowering phenotype of SWR1-C mutants, suggesting that the SWR1-C is needed to induce or regulate the *FLC* gene expression (Deal et al., 2007, Meagher et al., 2007). 

Moreover, the knockdowns of the H2A.Z genes display a phenotype similar to *swc6* and *arp6* [22]. Several findings suggest that the SWR1-C plays a role in the enrichment of H2A.Z at *MADS-AFFECTING FLOWERING 4 (MAF4)*, *MADS-AFFECTING FLOWERING 5 (MAF5)*, along with *FLC* loci [21,22,31]. Recently, SKIP protein in *Arabidopsis* was also shown to regulate the flowering time by directly binding to *SWC6* pre-mRNA and splicing it. However, in *skp*-1 mutants, the unregulated splicing of SWC6 leads to a decrease in H2A.Z enrichment at FLC chromatin, in turn activating the flowering [39]. Moreover, the interaction of *Arabidopsis* SWC4 and SWC6 govern the deposition of H2A.Z that ultimately brings the changes in growth and developmental processes. RNAi lines of SWC4 (*swc4i*) display changes in vegetative and reproductive phenotypes by altering the expression of key flowering genes. Flowering time in *swc4i* seems to be altered due to the increased expression of the floral integrator *FT* at the end of the daylight period under LD conditions, however, it does not affect the *FLC* locus or expression of *FLC*, which is reported to be controlled by SWR1-C, as discussed above [40]. Interestingly, a recent finding provides additional insight into flowering time regulation, showing that MUT9P-LIKE-KINASE (MLK4) mediates plant-specific phosphorylation of H2A at serine 95. This phosphorylation leads to the marking of nucleosome for H2A.Z enrichment at the *GIGANTEA (GI)* promoter. MLK4 gets recruited at the *GI* locus by CIRCADIAN CLOCK ASSOCIATED1 (CCA1) which also interacts with the YAF9a subunit of SWR1-C and NuA4 complexes, having an obligation for H2A.Z deposition and histone H4 acetylase activity, respectively [82]. This study relates phosphorylation of a single residue on histone H2A with the regulation of the flowering time. However, whether regulation could also be functional at different unknown loci those are participating in various physiological outcomes remains to be explored. 

Collectively, these observations indicate that SWR1-C assisted enrichment of H2A.Z is needed for sustenance of the vegetative phase growth, including floral repression through activation of *FLC* [55,80]. Though, these recent studies provide a better understanding of the flowering time regulation in plants. However, how these different components of SWR1-C are connected to other molecular switches that control several aspects of flowering is still obscure. Further studies will help us to understand the global connection and local arrangement of SWR1-C components during flowering in plants. 

## 7. H2A.Z Role in Response to Ambient Temperature

The growth and development of an organism requires optimum temperature, and variations in the optimum condition leads to an unfavorable stress condition. In plants, SWR1-C plays an indispensable role during ambient temperature signal perception. In response to warm temperatures, H2A.Z regulates the transcription of genes by affecting the ability of RNA Pol II [28]. A change in optimum temperature also functions as a key signal to other responses, such as a temperature-responsive change in chromatin structure was shown to be responsible for seed dispersal [83]. The authors showed that H2A.Z regulates the expression of the genes responsible for fruit dehiscence as a consequence of changes in optimum temperature [19,83]. Consistently, an increase in temperature decreases the levels of H2A.Z-nucleosomes at the *FT* promoter thereby facilitating the PHYTOCHROME INTERACTING FACTOR4 (PIF4) to bind and activate *FT* [84]. Moreover, the interaction of POWERDRESS (PWR) with HISTONE DEACETYLASE 9 (HDA9) is essential for temperature-induced hypocotyl/petiole elongation and early flowering [85,86]. Therefore, the association of H2A.Z with other chromatin modifications such as histone deacetylation, regulates gene expression to modulate developmental responses. Altogether, these findings contribute to a significant increase in our understanding of thermosensory H2A.Z dynamics [85]. 

## 8. Dual Role of H2A.Z

It is reported that the preferential enrichment of H2A.Z close to the transcription start site (TSS) facilitates the expression of various genes, however, increasing evidence indicates that the H2A.Z deposition also acts in the repression of gene expression possibly when it is localized in the gene bodies [24]. The repression could also happen due to H2A.Z deposition leading to repression of enhancer function along with the low gene accessibility at +1 nucleosomes for H2A.Z. Consistently, locus-specific deposition of H2A.Z by SWR1-C indicates that the enrichment of H2A.Z is distinctive for specific genes. In a study, the authors showed the distinctive deposition of H2A.Z by SWC4, which is achieved through sequence-specific recruitment of chromatin remodeling component on target genes for defined regulatory functions [40]. Additionally, a recent report in rice also supported preferential deposition of H2A.Z at the gene locus to negatively regulate nutrient-responsive genes, whereas the positive regulation was found for the expression of housekeeping genes during nutrient starvation [25]. In a different study, SWR1-C negatively regulated the expression of anthocyanin biosynthetic genes through enrichment of H2A.Z, which ultimately reduced the anthocyanin accumulation in *Arabidopsis* [34]. Moreover, another study suggests that the dual role of H2A.Z in gene expression depends on the post-translational modification of H2A.Z, whereas the H2A.Z level within the gene depends on the transcriptional activity [87]. The authors showed that the post-translational modification of H2A.Z is related to the transcriptional repression activity. H2A.Z, after getting ubiquitinated by PRC1 components *At*BMI1A/B/C, mediated the transcriptional repression activity independently from PCR2 [87]. Intriguingly in another study, the acetylation of histones at distinct sites was found to be responsible for the deposition of H2A.Z and regulation of DNA demethylation [9]. Nie et al. showed that ARP6 and PIE1 restrict the DNA hypermethylation and gene silencing, suggesting that H2A.Z deposition by SWR1-C is responsible for DNA demethylation. They also hypothesized that the deposition of H2A.Z is dependent on active histone acetylation marks, generated by increased DNA methylation (IDM) complex. These modified histones are recognized by MBD9 and NPX1 and thereby guide SWR1-C to these distinct sites to deposit H2A.Z, and finally H2A.Z recruits REPRESSOR OF SILENCING1 (ROS1) to prevent DNA methylation and gene silencing [9]. The preferential binding of MBD9 at the regions with active histone marks, and its interaction with SWR1-C, is also in agreement with the recent reports [9,56,57].

Despite the progress in our understanding of SWR1-C functions, several questions remain unanswered because of its diverse roles. Several missing links of SWR1-C regulation and the molecular switches involved in the process still need to be investigated.

## 9. Conclusions

In recent years, the research on chromatin remodeling complex, SWR1-C, has received considerable attention from researchers. Interestingly, we have acquired enormous progress and could answer some of the key questions of SWR1-C regulation. The role of several intermediate components, such as MBD9, in H2A.Z deposition by SWR1-C at a specific locus is becoming clear day by day. It could be expected that the number of such intermediates will increase rapidly in the near future, which could further increase our understanding of the underlying molecular principle and SWR1-C coordination with other interacting partners. The functional characterization of these partners in context to locus specificity may give us more information about SWR1-C mediated regulation of specific loci, while controlling global changes at the transcriptional level during optimal and adverse growth conditions. In any case, research on SWR1-C/H2A.Z is currently progressing, and exciting new knowledge is waiting to be explored. This acquired understanding of the SWR1-C function could be utilized as the basis for enhancing the tolerance of crop plants, particularly in the context of unfavorable conditions.

## Figures and Tables

**Table 1:** Summary of the responses (physiological, developmental, and environmental) mediated by SWR1C in *Arabidopsis*.

SWR1-C Subunit	Functions	References
PIE1	Regulation of flowering time, flower architecture, DNA damage repair, curly leaves, loss of apical dominance, immunity response, homeostasis of H3K27me3, regulation of anthocyanin biosynthesis, restriction of transgene silencing, regulation of gene expression	[9,20,29,30,31,32,33,34]
ARP6	Regulation of flowering time, flower architecture, DNA damage repair, regulation of cell cycle, defects in chromosome pairing and organization during female meiosis I, MMC specification, reduced fertility and seed set, inflorescence architecture, curly leaves, loss of apical dominance, immunity response, vegetative phase change, regulation of anthocyanin biosynthesis, phosphate starvation, restriction of transgene silencing, regulation of gene expression	[9,21,24,26,31,35,36,37,38]
SEF	Regulation of flowering time, flower architecture, loss of apical dominance, immunity response, vegetative phase change, inflorescence architecture, regulation of gene expression	[29,30,31,36,38,39,40]
YAF9	Regulation of flowering time and gene expression	[23]
SWC4	Sequence-specific recruitment of chromatin remodeling component, regulation of gene expression	[40]

**Table 2:** Yeast SWR1 sub-units and their homologues in *Arabidopsis.*

*S. cerevisiae*	*A. thaliana*	Locus
**Swr1**	PIE1	At3g12810
**ARP4**	ARP4	At1g18450
**ARP6**	ARP6	At3g33520
**YAF9**	YAF9A/TAF14B YAF9B/TAF14	At5g45600 At2g18000
**RVB1**	TIP49a/RIN1	At5g22330
**RVB2**	RVB2A RVB2B	At5g67630 At3g49830
**SWC2**	SWC2	At2g36740
**SWC3**	-	-
**SWC4**	SWC4	At2g47210
**SWC5**	SWC5	At5g30490
**SWC6**	SEF	At5g37055
**SWC7**	-	-
**ACT1**	ACT1	At2g37620
**BDF1**	-	-

## Supplementary Materials

The following are available online at https://www.mdpi.com/2073-4409/8/12/1621/s1, Figure S1: Summary of physiological and developmental responses mediated by SWR1-C.
