# Supplementary material for: SWR1 Chromatin Remodeling Complex: A Key Transcriptional Regulator in Plants
Source: Cells. 2019 Dec 12;8(12):1621. doi: 10.3390/cells8121621 (PMC6952815; doi:10.3390/cells8121621)
Supplement: Supplementary file 1 [file cells-08-01621-s001.pdf]

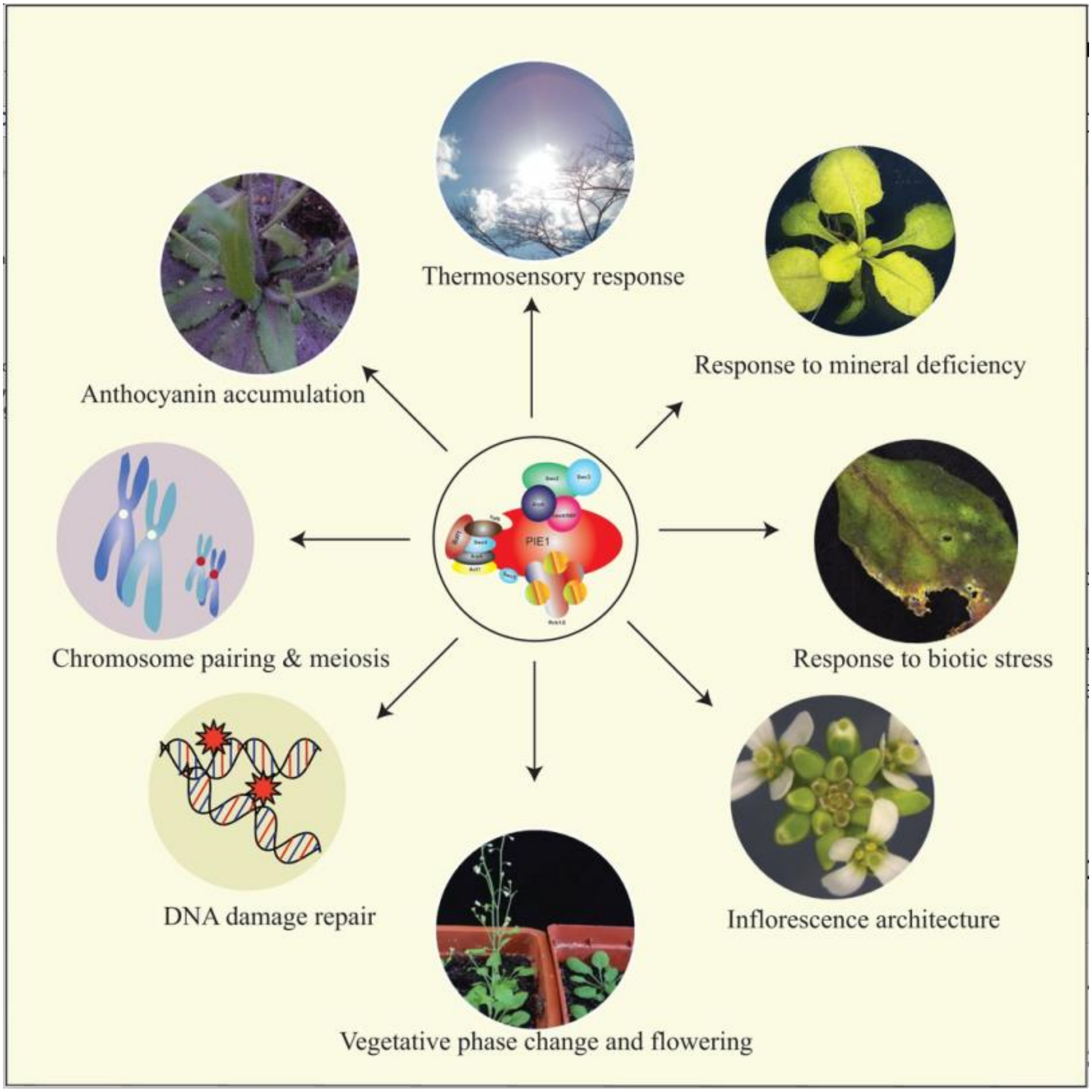

Supplementary Figure S1. Summary of physiological and developmental responses mediated by SWR1-C.
